# Supplementary material for: Effects of fecal microbiota transfer on blood pressure in animal models: A systematic review and meta-analysis
Source: PLoS One. 2024 Apr 5;19(4):e0300869. doi: 10.1371/journal.pone.0300869 (PMC10997129; doi:10.1371/journal.pone.0300869)
Supplement: S1 Table — (DOCX) [file pone.0300869.s002.docx]

**Table S1 Search strategy for each database**

| **Database** | **Search strategies** | **Last search date** | **Search results** |
| --- | --- | --- | --- |
| **PubMed** | (((((((blood pressure) OR (high blood pressure)) OR (hypertension)) OR (systolic blood pressure)) OR (SBP)) OR (diastolic blood pressure)) OR (DBP)) AND ((((((((((((((((((Fecal microbiota transplantation) OR (Fecal microbiota transfusion)) OR (Fecal transplantation)) OR (FMT)) OR (stool microbiota transplantation)) OR (stool microbiota transfusion)) OR (gut flora)) OR (intestinal flora)) OR (bacteriotherapy)) OR (fecal therapy)) OR (Fecal bacteriotherapy)) OR (Intestinal microbiota transplantation)) OR (fecal transplant)) OR (fecal transfusion)) OR (fecal implantation)) OR (fecal implant)) OR (fecal instillation)) OR (fecal reconstitution)) | **August 19, 2022** | **2199** |
| **Web of science** | #1 (((((((((((((((((TS=(Fecal microbiota transplantation)) OR TS=(Fecal microbiota transfusion)) OR TS=(Fecal transplantation)) OR TS=(FMT)) OR TS=(stool microbiota transplantation)) OR TS=(stool microbiota transfusion)) OR TS=(gut flora)) OR TS=(intestinal flora)) OR TS=(bacteriotherapy)) OR TS=(fecal therapy)) OR TS=(Fecal bacteriotherapy)) OR TS=(Intestinal microbiota transplantation)) OR TS=(fecal transplant)) OR TS=(fecal transfusion)) OR TS=(fecal implantation)) OR TS=(fecal implant)) OR TS=(fecal instillation)) OR TS=(fecal reconstitution)  #2 ((((((TS=(blood pressure)) OR TS=(high blood pressure)) OR TS=(hypertension)) OR TS=(Systolic blood pressure)) OR TS=(SBP)) OR TS=(Diastolic blood pressure)) OR TS=(DBP)  #3 #1 AND #2 | **August 20, 2022** | **2276** |
| **Cochrane Library** | #1 MeSH descriptor: [Fecal Microbiota Transplantation] explode all trees  #2 (Fecal microbiota transfusion):ti,ab,kw OR (Fecal transplantation):ti,ab,kw OR (FMT):ti,ab,kw OR (stool microbiota transplantation):ti,ab,kw OR (stool microbiota transfusion):ti,ab,kw  #3 (gut flora):ti,ab,kw OR (intestinal flora):ti,ab,kw OR (bacteriotherapy):ti,ab,kw OR (fecal therapy):ti,ab,kw OR (Fecal bacteriotherapy):ti,ab,kw  #4 (Intestinal microbiota transplantation):ti,ab,kw OR (fecal transplant):ti,ab,kw OR (fecal transfusion):ti,ab,kw OR (fecal implantation):ti,ab,kw OR (fecal implant):ti,ab,kw  #5 (fecal instillation):ti,ab,kw OR (fecal reconstitution):ti,ab,kw  #6 #1 OR #2 OR #3 OR #4 OR #5  #7 MeSH descriptor: [Blood Pressure] explode all trees  #8 (high blood pressure):ti,ab,kw OR (hypertension):ti,ab,kw OR (Systolic blood pressure):ti,ab,kw OR (SBP):ti,ab,kw OR (Diastolic blood pressure):ti,ab,kw  #9 DBP  #10 #7 OR #8 OR #9  #11 #6 AND #10 | **August 21, 2022** | **211** |
| **EBMSAE** | #1 'fecal reconstitution' OR 'fecal instillation' OR 'fecal implant' OR 'fecal implantation' OR 'fecal transplant' OR 'fecal transfusion' OR 'fecal microbiota transplantation' OR 'fecal microbiota transfusion' OR 'fecal transplantation' OR 'fmt' OR 'stool microbiota transplantation' OR 'stool microbiota transfusion' OR 'gut flora' OR 'intestinal flora' OR 'bacteriotherapy' OR 'fecal therapy' OR 'fecal bacteriotherapy' OR 'intestinal microbiota transplantation'  #2 'blood pressure' OR 'high blood pressure' OR 'hypertension' OR 'systolic blood pressure' OR 'SDB' OR 'diastolic blood pressure' OR 'DBP'  #3 #1 AND #2 | **August 22, 2022** | **3078** |
| **China National Knowledge Infrastructure** | SU='粪微生态移植'+'粪菌移植'+'粪便移植'+'肠道微生物移植'+'粪菌治疗'+'肠微生态移植'+'肠菌移植'+'肠道菌群移植'+'粪便细菌治疗'+'粪便微生物群移植' AND FT='血压' +'收缩压'+'舒张压' | **August 18, 2022** | **124** |
| **Weipu database** | U=(粪微生态移植 OR 粪菌移植 OR 粪便移植 OR 肠道微生物移植 OR 粪菌治疗 OR 肠微生态移植 OR 肠菌移植 OR 肠道菌群移植 OR 粪便细菌治疗 OR 粪便微生物群移植) AND U=(血压 OR 收缩压 OR 舒张压) | **August 18, 2022** | **13** |
| **Wanfang database** | 全部: ((血压) or 全部: (收缩压) or 全部: (舒张压) ) and 主题: ((粪微生态移植) or 主题: (粪菌移植) or 主题: (粪便移植) or 主题: (肠道微生物移植) or 主题: (粪菌治疗) or 主题: (肠菌移植) or 主题: (肠微生态移植) or 主题: (肠道菌群移植) or 主题: (粪便细菌治疗) or 主题: (粪便微生物群移植)) | **August 18, 2022** | **343** |
| **SinoMed** | ("粪微生态移植"[常用字段:智能] OR "粪菌移植"[常用字段:智能] OR "粪便移植"[常用字段:智能] OR "肠道微生物移植"[常用字段:智能] OR "粪菌治疗"[常用字段:智能] OR "肠菌移植"[常用字段:智能] OR "肠微生态移植"[常用字段:智能] OR "肠道菌群移植"[常用字段:智能] OR "粪便细菌治疗"[常用字段:智能] OR "粪便微生物群移植"[常用字段:智能]) AND ("血压"[常用字段:智能] OR "收缩压"[常用字段:智能] OR "舒张压"[常用字段:智能]) | **August 18, 2022** | **11** |
