# Supplementary material for: Effects of fecal microbiota transfer on blood pressure in animal models: A systematic review and meta-analysis
Source: PLoS One. 2024 Apr 5;19(4):e0300869. doi: 10.1371/journal.pone.0300869 (PMC10997129; doi:10.1371/journal.pone.0300869)
Supplement: Attachment — Submitted filename: plos review.docx [file pone.0300869.s003.docx]

144 why frozen feces can cause high sbp and fresh feces can’t? and why type of feces was not the source of heterogeneity? The statement here is not reasonable, please explain further on this point.

Please explain more clinical significance for the results of the study? How to treat hypertension by FMT in human in the future?

Which type of feces and which kind of delivery of FMT influence hypertension more? Please explain further.

How to explain recent negative study by Piotr Konopelski, whose conclusion did not comply with your conclusion? Is that related to measurement, FMT delivery, feces type or other kind of confounding factors?

Do the results of the study have practical implications for future studies?
