# Supplementary material for: Effects of fecal microbiota transfer on blood pressure in animal models: A systematic review and meta-analysis
Source: PLoS One. 2024 Apr 5;19(4):e0300869. doi: 10.1371/journal.pone.0300869 (PMC10997129; doi:10.1371/journal.pone.0300869)
Supplement: Attachment — Submitted filename: Response to Reviewers.docx [file pone.0300869.s004.docx]

No. PONE-D-23-22042

Dear Editors and Reviewers:

Thanks for your letter and for the reviewer' comments concerning our manuscript entitled “Effects of fecal microbiota transfer on blood pressure in animal models: A systematic review and meta-analysis” (No. PONE-D-23-22042). Those comments are valuable for revising and improving our paper with important guiding significance. According to the reviewers’ comments, we have made extensive modifications to our manuscript to make our results convincing. In this revised version, changes to our manuscript were marked in yellow highlight. The point-by-point response to the comments is given below.

**Responses to editor-in-chief:**

**Comment 1:** Your manuscript presents a timely systematic review and meta-analysis on the effects of fecal microbiota transfer on blood pressure in animal models. While I commend your thorough literature review and well-structured meta-analysis, there are concerns regarding the low sample sizes and high heterogeneity which could affect the robustness of the meta-analysis results. I recommend that you further enhance the discussion by addressing these limitations and emphasizing potential clinical applications.

***Response:*** Thank you for giving me such valuable advice. In the discussion section, we have included a discussion on the type of feces and route of delivery, as well as supplemented the clinical significance of the study (on Page 11-12 of the revised manuscript). We sincerely hope that this revision will lead to a clearer text.

**Comment 2:** Throughout the manuscript, please refrain from using unnecessary abbreviations.

***Response:*** Thank you for your reminder. We strongly agree with your suggestions. We thoroughly checked the manuscript and removed unnecessary abbreviations throughout.

**Comment 3:** There are several typographical and grammatical errors present, most notably in the abstract. Kindly ensure these are corrected.

***Response:*** Thank you for your meticulous work. Your suggestions have proven to be very valuable to us. To improve English and grammar, we have asked native English speakers to revise the manuscript.

**Comment 4:** I suggest adding subtitles in the discussion section. This will provide clarity, particularly when distinguishing between the systematic review and the meta-analysis.

***Response:*** We sincerely appreciate the valuable comments. We have added subtitles in the Discussion section.

**Responses to Reviewer #1:**

**Comment 1:** Why frozen feces can cause high sbp and fresh feces can’t? and why type of feces was not the source of heterogeneity? The statement here is not reasonable, please explain further on this point.

***Response:*** I am sorry that this part was not clear in the original manuscript. In this study, sub-group analysis was conducted based on the type of feces, and the meta-analysis results showed that in the fresh subgroup, there was no statistically significant difference between feces from hypertensive donors and feces from normotensive donors (MD=9.43, 95%CI: -3.12~21.99, P=0.140; *I^2^*=93). In the frozen subgroup, there was a statistically significant difference between feces from hypertensive donors and feces from normotensive donors (MD=21.50, 95%CI: 12.69~30.31, *P*<0.001; *I^2^*=96). However, both subgroups had high heterogeneity, so the type of feces is not the source of heterogeneity. In the results section (on Page 6-7, line 142-145 of the revised manuscript), we have corrected any incorrect statements. Only two trials used fresh fecal transplantation, and the meta-analysis of the combined results of the two groups showed no significant differences. The reason may be related to the small sample size and differences in blood pressure monitoring methods in the two trials. Due to insufficient data, further experimental studies are needed to research the effectiveness of fresh and frozen FMT.

**Comment 2:** Please explain more clinical significance for the results of the study? How to treat hypertension by FMT in human in the future?

***Response:*** Joanne et al.'s study summarized the findings on the involvement of the gut microbiota in the host's blood pressure regulation mechanism, and found that gut microbiota imbalance, characterized by a decrease in beneficial bacteria, can affect the host's microbiota-related gene pathways, leading to hypertension. The research discovered that fecal microbiota transplantation from hypertensive donors can lead to an increase in blood pressure in the recipient, suggesting a potential transfer of the hypertensive phenotype through the gut contents. Our study confirmed a causal relationship between hypertension and the gut microbiota, indicating that FMT may have potential therapeutic implications for hypertension. In the future, precise modulation of the gut microbiota through FMT and supplementation of beneficial gut metabolites may be used for hypertension treatment. However, further research is needed to determine the optimal fecal type and delivery method for human FMT. This may involve studying the effects of different donor types (such as healthy individuals or hypertensive patients) and administration routes (such as oral or colonoscopy) on blood pressure regulation. We have added content about the clinical significance of the research in the discussion section on Page 11-12 of the revised manuscript.

References:

1. O'Donnell JA, Zheng T, Meric G, et al. The gut microbiome and hypertension. Nat Rev Nephrol. 2023,19(3):153-167.
2. Zhong HJ, Zeng HL, Cai YL, et al. Washed Microbiota Transplantation Lowers Blood Pressure in Patients With Hypertension. Front Cell Infect Microbiol. 2021, 11:679624.

**Comment 3:** Which type of feces and which kind of delivery of FMT influence hypertension more? Please explain further.

***Response:*** In this systematic review, only two studies used fresh fecal, while the majority used frozen fecal bacteria. Frozen feces may offer benefits compared to fresh feces in terms of FMT preparation, storage, monitoring, and delivery ^(1)^. Quantitative polymerase chain reaction analyses have demonstrated that frozen and lyophilized FMT products can be stored for up to 7 months without any alteration in microbial composition or therapeutic efficacy ^(2)^. Previous study has indicated that frozen fecal transplant is more effective than fresh fecal transplant for ulcerative colitis, due to the reduction of gram-negative bacteria in frozen FMT ^(3)^. In this study, the transplantation of frozen fecal bacteria had a significant impact on the recipient's blood pressure, while the impact of fresh fecal bacteria was not significant. The specific reasons for this are unclear and should be further analyzed in future studies. The route of delivery may also be a key factor affecting the effectiveness of FMT. The delivery methods used in the experiments included in this study are oral gavage, oral inoculate, and intracolonic administration. Oral administration is the most common route for studies on rodents, as it is relatively simple. In our study, only one study used colonic administration, and the meta-analysis showed that oral administration had a greater impact on blood pressure. Therefore, the impact of different delivery methods on blood pressure may vary for different recipients, and targeted research is still needed.

References:

1. Lee CH, Steiner T, Petrof EO, et al. Frozen vs Fresh Fecal Microbiota Transplantation and Clinical Resolution of Diarrhea in Patients With Recurrent Clostridium difficile Infection: A Randomized Clinical Trial. JAMA 2016, 315:142-9.
2. Jiang ZD, Alexander A, Ke S, et al. Stability and efficacy of frozen and lyophilized fecal microbiota transplant (FMT) product in a mouse model of Clostridium difficile infection (CDI). Anaerobe 2017;48:110-4.
3. Zhu F, Ke Y, Luo Y, Wu J, Wu P, Ma F, Liu Y. Effects of Different Treatment of Fecal Microbiota Transplantation Techniques on Treatment of Ulcerative Colitis in Rats. Front Microbiol. 2021, 12:683234.

**Comment 4:** How to explain recent negative study by Piotr Konopelski, whose conclusion did not comply with your conclusion? Is that related to measurement, FMT delivery, feces type or other kind of confounding factors? Do the results of the study have practical implications for future studies?

***Response:*** In the study by Piotr Konopelski et al., it was found that an inter-strain fecal transplant does not affect blood pressure and does not produce long-term changes in gut bacteria composition, which is contrary to the conclusions of our study. We speculate that the reason for this is that in this study, blood pressure in mice was measured using telemetry recordings, fecal transplantation was administered via colonic gavage using fresh fecal material, which differs significantly from the other experiments included in our study. It would be beneficial to replicate this study design in the future and validate the experimental results. Our study results emphasize the potential clinical application of FMT in the management of hypertension, but further research is needed to determine the optimal strategy for implementing FMT in humans. This study confirmed a causal relationship between hypertension and the gut microbiota, indicating that FMT may have potential therapeutic implications for hypertension. In the future, precise modulation of the gut microbiota through FMT and supplementation of beneficial gut metabolites may be used for hypertension treatment. However, further research is needed to determine the optimal fecal type and delivery method for human FMT. This may involve studying the effects of different donor types (such as healthy individuals or hypertensive patients) and administration routes (such as oral or colonoscopy) on blood pressure regulation

We sincerely hope that this manuscript has revised all your comments and suggestions, which we marked in highlight in our changes. We appreciate for editors' and reviewers’ warm work earnestly and hope the correction will meet with approval. We will be happy to edit the manuscript further, based on helpful comments from the reviewers.

Once again, thank you very much for your comments and suggestions.

Best wishes,

Yanjuan Lin
