# Supplementary material for: Effects of fecal microbiota transfer on blood pressure in animal models: A systematic review and meta-analysis
Source: PLoS One. 2024 Apr 5;19(4):e0300869. doi: 10.1371/journal.pone.0300869 (PMC10997129; doi:10.1371/journal.pone.0300869)
Supplement: Attachment — Submitted filename: Response to Reviewers.docx [file pone.0300869.s005.docx]

No. PONE-D-23-22042R1

Dear Editors and Reviewers:

Thanks for your letter and for the reviewer' comments concerning our manuscript entitled “Effects of fecal microbiota transfer on blood pressure in animal models: A systematic review and meta-analysis” (No.PONE-D-23-22042R1). Those comments are all valuable and very helpful for revising and improving our paper, as well as the important guiding significance to our researches. We have studied comments carefully and have marked in the yellow highlight in the paper. The point-by-point response to the comments is given below.

**Responses to Reviewer #2:**

**Comment 1:** The article presents results from subgroup analyses based on blood pressure measurement methods, fecal material type (frozen vs. fresh), and route of delivery, and animal model. Can the authors elaborate on the rationale behind selecting these specific subgroups for analysis?

***Response:*** I am sorry that this part was not clear in the original manuscript. The implementation process of FMT has not reached a consensus, and factors such as fecal material type (frozen vs. fresh), and route of delivery may affect the results. To explore the sources of heterogeneity in research results and provide evidence for the clinical implementation of FMT, this study conducted subgroup analyses on possible influencing factors.

Studies have shown that the storage conditions of fecal samples can affect the composition of the microbiota. European consensus conference on faecal microbiota transplantation in clinical practice indicates that fresh feces within 6 hours should be used; before further preparation, feces should be stored in sealed containers at 2-8°C (Quality of evidence: moderate. Strength of recommendation: strong). Preparation of frozen feces (Quality of evidence: moderate. Strength of recommendation: strong). Frozen transplant materials can simplify the microbiota transplantation process without reducing efficacy and safety. Our study found that the use of frozen fecal microbiota transplantation had a greater impact on blood pressure. There are many routes for fecal microbiota transplantation, mainly divided into oral and nasal (nasogastric or capsule ingestion) or colonic implantation. The oral and nasal route is widely used and easy to implement. Colonic implantation is consistent with the recipient's physiology, but there is an issue that fecal liquids have a short retention time in the gastrointestinal tract. Currently, there is no clear evidence to support which transplantation route is most appropriate. In our manuscript, there were more studies using the oral route than the colonic implantation, and the results showed that the oral route had a greater impact on recipient blood pressure.

The blood pressure measurement methods included tail-cuff blood pressure system and carotid artery direct register. Carotid artery direct register is directly transmitted arterial pressure waveforms through sensors, while tail-cuff blood pressure system measures blood flow within arteries. Differences in measurement methods could lead to differences in results. Therefore, we conducted subgroup analyses based on blood pressure measurement. The studies included in our manuscript involved subjects including both normal blood pressure and hypertensive subjects. We also conducted subgroup analyses based on animal models (normotensive model and hypertension model) to explore the effects of fecal microbiota transplantation on subjects with normal blood pressure and hypertensive subjects, respectively.

In the discussion section, we have briefly supplemented the reasons for conducting subgroup analysis (on Page 10-11 of the revised manuscript). We sincerely hope that this revision will lead to a clearer text.

**References:**

1. European consensus conference on faecal microbiota transplantation in clinical practice[J].Gut Journal of the British Society of Gastroenterology, 2017.
2. Cammarota,Giovanni,Ianiro,et al.Faecal microbiota transplantation in clinical practice[J].Gut Journal of the British Society of Gastroenterology, 2018.

**Comment 2:** The visual inspection of funnel plots is a common method to assess publication bias, but it is somewhat subjective. Did you consider complementing this with more objective statistical tests, such as Egger's test or Begg's test, to provide a more robust analysis of publication bias?

***Response:*** Thank you for your reminder. We strongly agree with your suggestions. We used the Egger’s and Begg’s test of STATA version 12.0 to assess the possibility of publication bias. Egger’s and Begg’s test shows that p value was higher than 0.05 (p for Begg’s test = 0.536; p for Egger’s test = 0.190), indicating that there was no significant publication bias observed in the selected studies (Figure 3). In the results section (on Page 6, line 127-129 of the revised manuscript), we have rephrased the publication bias results.

**Comment 3:** Have you ever thought that the dosage of fecal infusion might influence the outcome? Although Zhang et al.’s study in 2020 (https://doi.org/10.1007/s13238-019-00684-8) ever reported that the fecal weight was not well correlated with the amount of enriched microbiota, what results caused by dosage of fecal infusion do you think your research will bring?

***Response:*** We agree with your viewpoint that the dosage of fecal microbiota transplantation would affect the results. We conducted a subgroup analysis based on the dosage of fecal microbiota transplantation, and the results showed that transplantation of fecal bacteria from the hypertensive model led a significant increase in SBP (MD=18.55, 95%CI=6.23-30.87, *P*=0.003; *I^2^*=98%) in the 1ml fecal contents subgroup, but the sensitivity analysis results did not change after excluding studies one by one. The 750µl fecal contents subgroup showed similar results (MD=19.15, 95%CI=12.84-25.47, *P*<0.001; *I^2^*=0). The single study in the 250µl fecal contents subgroup also demonstrated comparable results (MD=15.81, 95%CI=11.26-20.36, *P*<0.001; *I^2^*=96%). In the results section (on Page 7-8, line 164-171 of the revised manuscript), we have rephrased the publication bias results.

In the discussion section, we have discussed the impact of fecal microbiota dosage on the results (on Page 11 of the revised manuscript). The amount of feces does not have a proportional relationship with the number of microbes, and there are large individual differences. Currently, there is no unified standard for the amount of feces collected. A larger transplantation dose can increase the number and types of donor microbes in the subject's gut while prolonging the stability of probiotics. However, the increased microbial diversity may also produce various metabolic products and damage the intestinal immune barrier, thereby reducing the effectiveness of FMT. Therefore, more well-designed RCTs or stratified cohort studies are still needed to evaluate the impact of different doses. Considering the total transplantation dose may also affect the results of FMT, the combined design of dose and frequency can also be included in future research objectives.

In the manuscript, we have removed the 8th reference because it is not covered in SCI-indexed journals and it does not affect the overall content of the article (Fang H, Fu L, Wang J. Protocol for Fecal Microbiota Transplantation in Inflammatory Bowel Disease: A Systematic Review and Meta-Analysis. Biomed Res Int. 2018:8941340.).

We sincerely hope that this manuscript has revised all your comments and suggestions, which we marked in highlight in our changes. We appreciate for editors' and reviewers’ warm work earnestly and hope the correction will meet with approval. We will be happy to edit the manuscript further, based on helpful comments from the reviewers.

Once again, thank you very much for your comments and suggestions.

Best wishes,

Yanjuan Lin
